# Evaluation of the Depth and Width of Cuts after Controlled-Depth Abrasive Water Jet Machining Using Low Pressure

**DOI:** 10.3390/ma16247532

**Published:** 2023-12-06

**Authors:** Frantisek Botko, Dominika Botkova, Matus Gelatko, Radoslav Vandzura, Dagmar Klichova

**Affiliations:** 1 Faculty of Manufacturing Technologies, Technical University of Košice with Seat in Prešov, 080 01 Prešov, Slovakia; 2 The Czech Academy of Sciences, Institute of Geonics, 708 00 Ostrava, Czech Republic

**Keywords:** abrasive water jet, controlled-depth machining, depth of cut, erosion track

## Abstract

The presented paper is focused on the evaluation of material removal during machining via an abrasive water jet with a controlled depth of cut. In the introductory parts of the work, a theoretical analysis of water jet technology and an analysis of the current state of the problem are presented. The experimental part of the work is devoted to testing the effects of technological parameters on material removal from the point of view of the maximum erosion depth and volume loss of material during machining with a low water pressure of 50 MPa. The tested material was a Ti 6Al 4V titanium alloy. The experiments were carried out by changing the traverse speed of the cutting head, the mass flow of the abrasive and the angle of inclination of the cutting head, according to the DoE 3^3^ experiment plan. The obtained values were evaluated using the method of variance (ANOVA) and regression analysis. Furthermore, the values of the width of the erosion track and the maximum and minimum erosion effects for both tested materials were evaluated.

## 1. Introduction

Currently, great importance is placed on the innovation and improvement of various technologies and processes, and on the search for new alternative ways of approaching the production of semi-finished products and finished products. In industry, the application framework of difficult-to-machine materials is expanding due to their unique properties, such as their high strength, high corrosion resistance, heat resistance, etc. From this point of view, it is necessary to search for optimal processing processes for these materials with regard to the economy of the process, the quality of the newly created surfaces, and, last but not least, the environmental impact of the production process.

The application of abrasive water jet machining (AWJ) with a controlled depth of cut creates possibilities for the machining of difficult-to-machine materials. The advantages of this technology include the absence of a physical rigid body as a tool, as the function of the tool is performed by a focused stream of water and abrasive particles. Such a tool is not subject to wear and tear in the machining process, as a new tool with the required properties is in use at every moment. Other positives include the small diameter of the “tool”, at the level of ± 1 mm, and minimal lateral forces during machining, which makes it possible to produce thin-walled components with a smaller wall thickness compared to chip machining methods. The absence of a heat-affected zone for practically all industrial materials is also a great benefit brought about by AWJ technology. When AWJ machining with a controlled depth of cut, there is no need to change the tool, since, by changing the technological conditions, it is possible to change the material removal conditions continuously, online, without the need for technological downtime. From an environmental point of view, AWJ technology is characterized by the absence of process media in the form of cutting oils and emulsions, and it has acceptable energy requirements when using lower pressures.

The present work is aimed at determining the influence of the technological conditions of the AWJ, during machining with a controlled depth of cut, on the removal characteristics (erosion depth, volume loss of material, and width of the trace after the AWJ transition) of difficult-to-machine titanium alloy Ti 6Al 4V. The methods of the planned experiment (DoE), an analysis of variance (ANOVA), and regression analysis are used to evaluate the effect of the technological conditions.

The velocity of the particles on their exit from the focusing tube can be calculated based on the work of Hashish [1,2].

In fact, the abrasive injection water jet is composed of three phases (solid–abrasive particles, liquid–water and gas–air) and it is possible to define the energy profile of the AIWJ (abrasive injection water jet) as a sum of the kinetic energies of the individual components of the jet. The energy distribution depends on the velocity and mass profile of the individual phases of the AIWJ. A predominant amount of kinetic energy is transferred by the abrasive particles and water. Despite its supersonic discharge velocity, the mass of air is minimal and thus it is possible to neglect the kinetic energy of air [3,4,5].

The energy available at the time interval *t_j_*, which belongs to the interval of the abrasives’ feed, is divided between the kinetic energy of the water jet (1) and the kinetic energy of the abrasive particles (2), according to Equations [3,4,5]:

(1) $$E_{KWJ,j}=\frac{m_{WJ,j}v_{WJ,j}^{2}}{2}$$

(2) $$E_{KAP,j\;}=E_{KAP,j}^{T}+E_{KAP,j}^{R}=\frac{m_{AP,j}v_{AP,j}^{2}}{2}+\frac{J_{AP,j}\omega_{AP,j}^{2}}{2}$$

where, for the time interval *t_j_*, the allocated values are:

*m_WJ,j_*—Mass of part of the water jet;
*v_WJ,j_*—Velocity of the water jet;
*m_AP,j_*—Mass of the abrasives;
*v_AP,j_*—Average velocity of the abrasives;
*ω_Ap,j_*—Angular velocity of the abrasives;
*J_AP,j_*—Inertia momentum of the abrasives.

The abrasive water jet is characterized by its dependence on the material removal rate (MMR) of the water pressure and the jet and abrasives’ velocities. The acceleration of abrasive particles occurs in the jet of water, so the pressure of water can be considered a prevalent parameter. The MMR thus depends on the power supplied by the abrasives. The process of groove creation is connected to energy loss via material removal. With an increasing penetration depth, a decrease in groove width can be observed. In general, it can be stated that grooves are wider on the upper erosion base than on the lower erosion base [4].

According to the results of Equation (2), the kinetic energy of the abrasive particles is defined as the sum of the translational and rotational components of the kinetic energy [5,6].

With a suitable combination of an AIWJ and the workpiece material’s properties, it is possible to apply the AIWJ to different specific operations in industry, which are difficult to achieve via conventional machining processes. One of the technological modifications that can be considered with AIWJ is controlled-depth machining (also called AWJ milling) [7,8,9]. AWJ milling is applicable to the machining of hard-to-machine materials, where a traditional milling process (a rotational tool with defined geometry of cutting edge) is technologically difficult and costly. Multiple authors have focused their research on AWJ milling [7,10]. At the early stages of AWJ controlled depth machining was the pioneer Mohamad Hashish, who studied machining on a wide spectrum of materials, such as aluminum alloys, titanium, and nickel alloys [11]. AWJ milling of carbon epoxy laminates was studied by Hejjaji et al. in 2017 [12] and 2019 [13], and machining of titanium alloys was examined by Kanthababu et al. (2016) [14] and Popan et al. (2016) [15]. Aluminum alloy AW 2024 was the subject of research by Cenac et al., 2015 [16]. Machining of the nickel superalloy Inconel was examined by Escobar-Palafox et al., 2012 [17], and composite materials were examined by Srinivasu and Axinite, 2014 [18]. A comparison of milling using AWJ for different types of steel (stainless, low carbon, and tool steel) was carried out by Gupta et al. (2013) [19]. The possibility of machining (milling) the tool steel using AWJ was examined by Pal et al. in 2013 [20]. In their research, Alberdi et al. (2010) [21] found out that in AWJ milling, the depth of the cut is directly dependent on traverse speed. For this reason, it is difficult to maintain a constant depth of cut during the machining process. Also, acceleration and deceleration of the cutting head influence the depth of the cut. In conclusion, they expressed the opinion that it is necessary to develop more advanced control strategies. These control strategies would be able to adapt the cutting conditions (pressure, abrasive mass flow) so that the effect of variable feed rate on the final quality of the machined surface could be optimized. Alberdi et al. (2010) formulated the opinion that advanced control strategies can be applicable to pocket machining [21]. The idea of pocket machining was also a subject of research conducted by Botko et al. (2020), which stated a similar opinion [22]. Other authors that deal with the problems of AWJ milling and slotting include Azarsa et al., (2020) [23], who studied micromachining of thin-walled structures with a high height/thickness ratio. Azarsa et al. (2020) [24] studied the influence of traverse speed, number of passes, pressure, spacing, and abrasive mass flow change on the final shape of the rib. Research shows that with a lower traverse speed, a wider groove between the ribs was created, but the generated channel was deeper and less influenced by suspension flow. Higher pressure causes an increase in flow and an increase in erosion. With lower traverse speed and higher pump pressure, they proved the creation of ribs with 500 μm spacing and height/thickness ratio > 40. Ribs with such parameters are problematic to manufacture by conventional machining processes; thus, in this area, the potential of AWJ milling is very high [23,24].

In AWJ machining, a liquid is supplied by a high-pressure pump to a multiplier where pressure is increasing. Pressurized water is then supplied to a high-pressure buffer, which ensures constant pressure in the system and dumping of strokes, which are created in the system. After exiting the nozzle orifice, a high-speed water jet flows through a mixing chamber where the mixing of the liquid phase (water), solid phase (abrasives), and part of the air sucked with the abrasives via the Venturi effect occurs, and thus the AWJ is created. An abrasive mixture then enters the focusing tube and subsequently exits the focusing tube, at which point it can be used as a tool [25].

The AWJ machining process (cutting or disintegration) is influenced by the hydraulic, cutting, mixing, and abrasive factors of the process. Also, the performance of the AWJ (depth of penetration, tilt angle, material removal rate, surface roughness) and surface integrity (topography and metallography) takes place [26].

Uhlmann et al. [27] described four approaches of controlled depth machining using AWJ: cutting, controlled depth milling, controlled depth cutting, and segment cut with a controlled depth of removal.

According to [28] Balc et al., AWJ milling strategies can be divided into five approaches: face milling, shape milling, line strategy, contour strategy, and milling using a mask. With an optimal machining strategy, it is possible to create plane surfaces, profiles, pockets, grooves, and thin-walled components out of metallic and non-metallic materials, using AWJ.

Holmberg et al. [29] elaborated a comprehensive study of nickel-based alloy Inconel 718 machining, using two devices with pressure ranges of 50–300 MPa (device 1) and 137.9–345 MPa (device 2). With a combination of machining parameters (traverse speed, pressure, and standoff distance), they described the depth and width of the erosion track for different combinations. Simultaneously, multi-pass machining and a combination of roughing (high pressure) and finishing (low pressure) passes were described [29]. Research into limestone cutting efficiency using a low-pressure (28 MPa) suspension water jet was performed by Perec [30]. The paper describes promising results of low-pressure machining in comparison with high-pressure applications. The application of an orthogonal array design for the optimization of suspension abrasive water jets was addressed by Perec [31]. The author discusses the incorporation of orthogonal array design methods into the research of abrasive suspension water jets.

The presented research was motivated by a lack of experimental data for AWJ-controlled depth machining with 50 MPa pump pressure, which shows potential in the machining of the wide spectrum of metallic and non-metallic materials.

## 2. Materials and Methods

**Figure 1:**
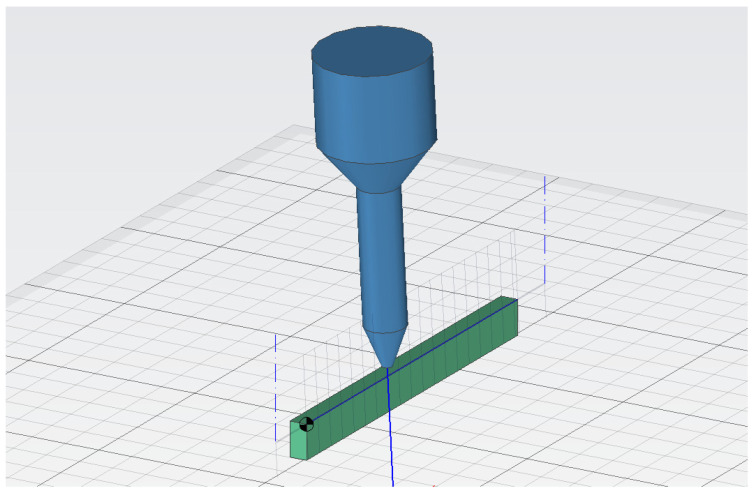
Preparation of the machining process.

The preparation of NC programs (Figure 1) for machining of experimental samples took place in the CAD software Igems R2021 (Teknikgatan 3, 504 62 Borås, Sweden), which is intended for creating programs for the entire range of energy beam cutting technologies, including oxygen, plasma, laser, and water jet. The software is user-friendly for the creation of simple programs and, at the same time, contains many functions for advanced technology management. The advantage is also the possibility of working in 3D, which allows us to efficiently program the tilting of the cutting head of the machine. The programs themselves can be created in two modes—machine control and software control. These two modes differ mainly in the possibility of changing the method, time of the shot, the choice of material, and the mass flow of the abrasive. During the programming in the machine control mode, the contour of the cut and the required quality of the cut (Q1–Q5) are defined, and after exporting the program to the control system of the machine, it is possible to change the thickness of the material, water pressure, strategy and time of the shot, mass flow of the abrasive for the shot, and cutting itself, while the machine’s control system recalculates the feed rate based on the specified cut quality Qx. For the presented experimental research, a custom data set was created, in which Qx presets were assigned to fixed values of traverse speed for each abrasives mass flow according to the experimental design DoE 3^3^ (for example, for *m_a_* = 20 g·min^−1^ Q1—100 mm·min^−1^; Q2—200 mm·min^−1^; Q3—300 mm·min^−1^). These values are not in compliance with standard SN 214001:2010, which defines the quality of the created surface, but are assigned desired factor levels during each run [32].

Experimental machining of titanium alloy Ti 6Al 4V (Table 1) was conducted using a Water Jet 3015 RT-3D (Kovostrojservis, spol. s.r.o, Pardubice, Czech Republic) with a dimension of 3000 mm × 1500 mm and a 3D cutting head with a 45° tilt on two axes. Pressurized water was generated using a PTV Jets 3.8/60 high-pressure pump, with maximal pressure of 415 MPa and a water flow volume of 3.8 dm^3^·min^−1^. Chemical composition and mechanical properties of TI 6Al 4V ale shown in Table 2. The abrasive used for experimental machining was Australian garnet GMA ClassicCutTM. 80 (GMA Garnet Group (Head Office). Level 4, Perth, Australia) (alluvial almandine garnet) with a grain size of 300–150 microns and a distribution of grain size of 300 microns—20%; 250 microns—32%; 212 microns—23%; 180 microns—14%; 150 microns—6% according to the data sheet [33].

**Figure 2:**
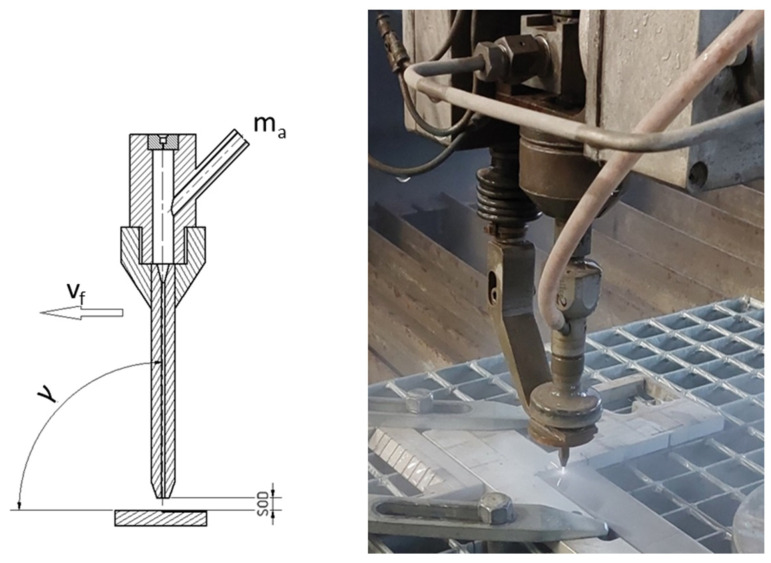
Schematics and realization of experiment.

Experiments were performed (Figure 2) with three variable technologic parameters (traverse speed of the cutting head, abrasive mass flow, and cutting head tilt) (Table 3), and the rest of the parameters were fixed (*p*—water pressure, *SOD*—standoff distance, *Dv*/*Df*—diameter of nozzle orifice/diameter of focusing tube) (Table 2).

Experiments were realized according to the design of experiments (Table 3) with 27 combinations of parameters with 3 repetitions. In total, 81 runs were realized (3 × 27).

**Figure 3:**
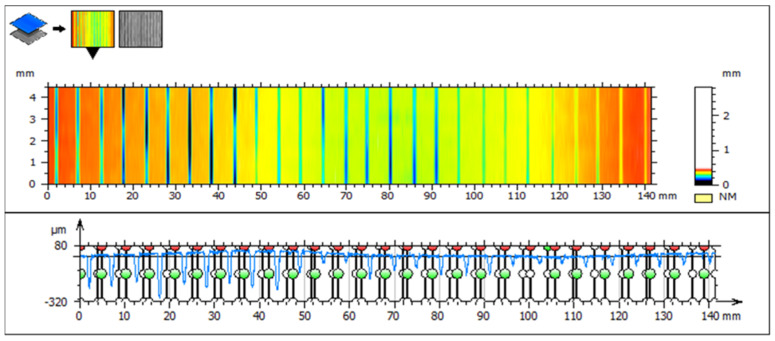
Visualization of erosion tracks on sample 1.

Observation of erosion track depth, created by one pass of AWJ for every combination of variable technological parameters, was realized using an optical profilometer MicroProof FRT. For each erosion track, measurement of 10 lines was performed, where the depth of the track and the surface of the crosscut were recorded. Hence, for each of the 27 combinations and 3 repetitions, 10 values were recorded (810 in total). Average values were calculated for every combination of variable factors (after excluding outliners by the Grubss test) and the Kolmogorov–Smirnov test of normal distribution of measured values was conducted. Subsequently, data were analyzed using ANOVA and regression analysis. The following figure (Figure 3) represents sample 1. In the image, visualization of the eroded grooves on the scanned surface of sample 1 can be observed. In the upper part of the image, the height change is distinguished by color, which shows the depth of the grooves and thus visualizes the erosion efficiency of the water jet. In the lower part, the depicted profile of all grooves shows the erosion efficiency. For the evaluation of the depth of each groove, the profile is divided into 27 sections.

## 3. Results

**Figure 4:**
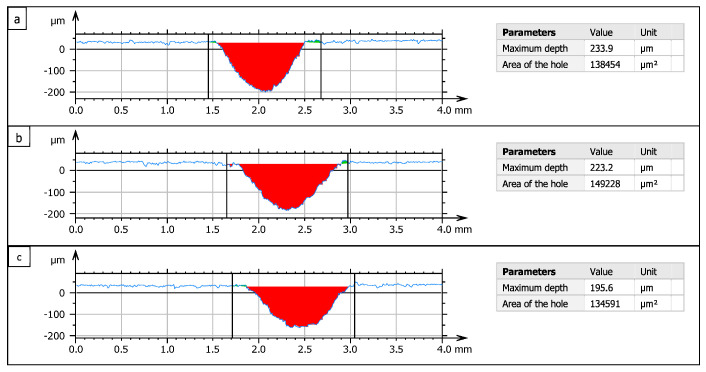
Visualization of erosion tracks crosscuts. *γ* = 90° (**a**), 92.5° (**b**), and 95° (**c**).

Profiles of erosion tracks in Ti 6Al 4V (Figure 4) created by one pass of AWJ at *v_f_* = 100 mm·min^−1^ and *m_a_* = 20 g·min^−1^ and tilt angle of cutting head *γ* = 90° (a), 92.5° (b), and 95° (c) show that when the cutting head is tilted, AWJ generates wider erosion tracks with milder inclination of the side walls compared to a perpendicular position of the cutting head.

Using analysis of variance (ANOVA) (Table 4), variable technological parameters (factors) from the perspective of their statistical significance on the observed dependent variable were verified.

Considering the fact that all values of the *p*-test criteria are lower compared to significance level α = 0.05, it is possible to accept null hypothesis H0, which assumes that the selected model covers the variability of the dependent variable response.

*a_pTi_ =* 810.9 − 4.399*γ* + 1.162*m_a_* − 2.366*v_f_* + 0.003792*v_f_*^2^ (3)

The regression Equation (3) describes the influence of AWJ technological parameters on observed response ap (depth of erosion track, thus the depth of cut) for the material Ti 6Al-4V. The coefficient of determination R^2^ is 0.9666, which means that 96.66% of process variability can be explained by the stated regression model. Hence, the regression model is suitable for describing presented dependence. The adjusted coefficient of determination R^2^U = 0.9649 proves the high significance of the regression model.

**Figure 5:**
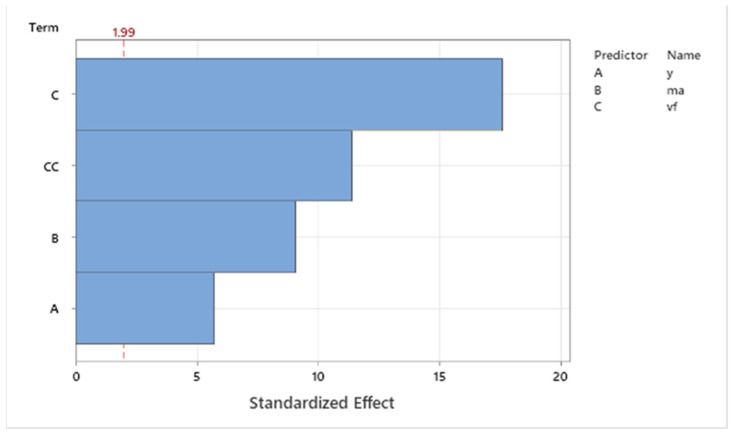
Pareto graph for *a_p_*.

Pareto analysis (Figure 5) shows the degree of influence of single technologic parameters (*v_f_*, *m_a_*, and *γ*) on the observed values of the dependent variable *a_p_*. The critical value for the inclusion of single factors into the regression model was 1.99. As is evident from the Pareto graph, the most significant factor is the traverse speed of the cutting head, which is followed by the first-level power interaction of the traverse speed. The next significant factors according to ANOVA were abrasive mass flow and the tilt angle of the cutting head. Based on the obtained results, the high significance of all factors can be stated, including the resulting regression equation.

**Figure 6:**
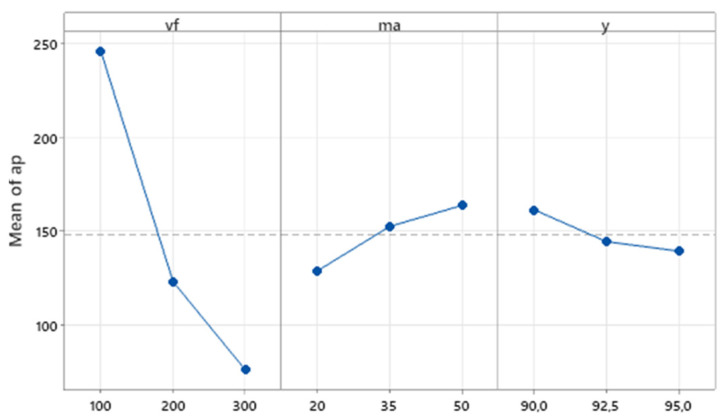
Main effect plot for *a_p_*.

The graph in Figure 6 shows the influence of the main effects of independent variables on the average value of the dependent variable. The main effects plot shows that the biggest influence on the resulting depth of erosion track has the traverse speed of the cutting head (*v_f_*), where for traverse speed 100 mm·min^−1^, a 250 µm depth of the erosion track can be observed, for a medium level of *v_f_* , the depth is 130 µm, and for 300 mm·min^−1^, it is *a_p_* 70 µm. The effect of the other two technological factors on the values of the dependent variable is considerably lower in the range of values applied in the presented research, but their influence is still statistically significant. For abrasive mass flow *m_a_*, a rising trend of *a_p_* can be observed, with increasing values of ma in the range of 20–50 g·min^−1^. Conversely, for the tilt of the cutting head, *γ* can be observed as a descending trend with the rising tilt angle.

**Figure 7:**
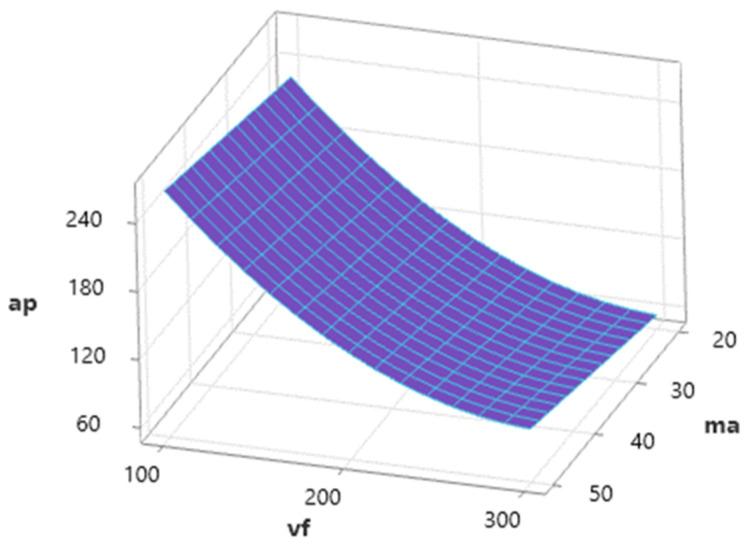
Dependence of *a_p_* on *v_f_* and *m_a_* (*γ* = 92.5°).

Figure 7 shows the influence of independent variables (*v_f_* and *m_a_*) on dependent variable *a_p_* for a medium value of *γ* = 92.5°. Dependence shows a high influence of *v_f_* and, with an increase in this factor, a significant decrease in ap values is observed. Conversely, abrasive mass flow has a positive influence on *a_p_,* and this factor acts to increase *a_p_* values.

**Figure 8:**
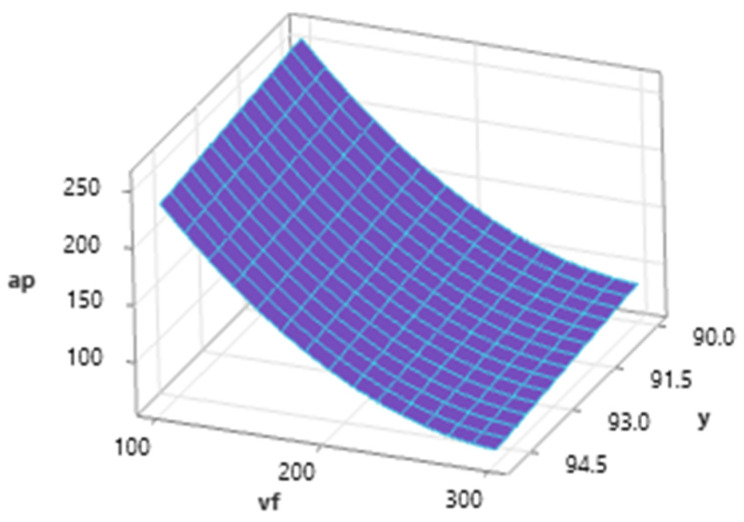
Dependence of *a_p_* on *v_f_* and *γ* (*m_a_* = 35 g·min^−1^).

Figure 8 shows the influence of traverse speed and tilt angle with a medium level of abrasives mass flow (*m_a_* = 35 g·min^−1^) on resulting *a_p_* values. The dependence shows a significant influence of the factor *v_f_* on the achieved depth of the erosion track, and therefore the depth of the cut. The effect of the tilt of the cutting head is lower, and during the increasing tilt angle from the perpendicular, there is a slight decrease in the maximum depth of the erosive track at the same *v_f_* value.

**Figure 9:**
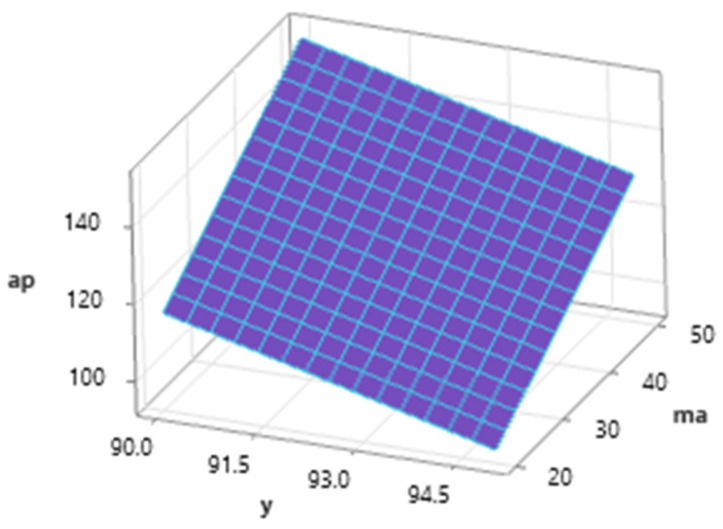
Dependence of *a_p_* on *γ* and *m_a_* (*v_f_* = 200 mm·min^−1^).

The graph of the dependence (Figure 9) of the depth of cut ap on the mass flow of the abrasive and the inclination angle of the cutting head at the mean value of *v_f_* (200 mm·min^−1^) shows a positive trend of ap when increasing the m_a_ value from 20 to 50 g·min^−1^ and a negative trend of the *a_p_* value when the cutting head tilts away from the vertical. The maximum value was reached for a feed speed of 200 mm·min^−1^ at the combination of *m_a_* = 50 g·min^−1^ and *γ* = 90° and the minimum at *m_a_* = 20 g·min^−1^ and *γ* = 95°.

∆*V_Ti_* = 0.4805 − 0.001196*v_f_* + 0.002353*m_a_* − 0.00235*γ* + 0.000002*v_f_*^2^ (4)

The results of ANOVA are listed in Table 5. The regression Equation (4) describes the effect of the technological parameters of AWJ on the monitored response Δ*V* (volume loss) for the Ti 6Al-4V material. The coefficient of determination of the R^2^ model reaches a value of 0.8812, which means that 88.12% of the variability can be explained by the given model. The model is therefore suitable for describing the given dependence. The adjusted coefficient of determination R^2^U = 0.8750 testifies to the high significance of the model.

**Figure 10:**
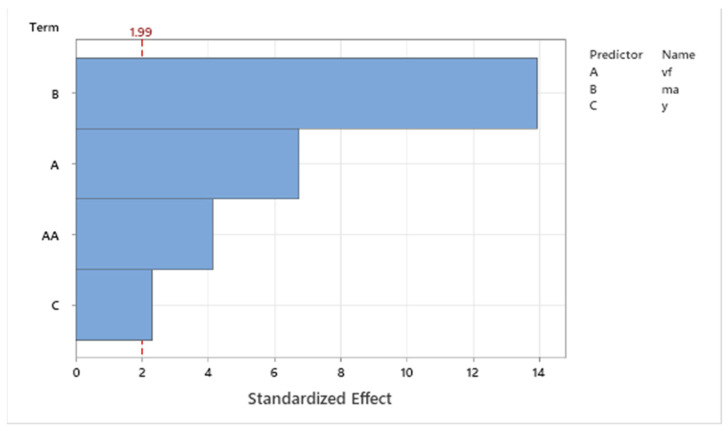
Pareto graph for Δ*V*.

Pareto analysis (Figure 10) of the standardized effects of the input variables of the technological conditions on the monitored response Δ*V* shows that in the case of the material Ti 6Al 4V, the abrasive has a high influence on the mass flow, the effect of which has a value of 14. The second most significant factor of Ti is the feed speed of the cutting head, the value of which reached 7, followed by a power interaction of the same factor, where an effect of 4 was observed. The least significant factor for the observed response was the tilt angle of the cutting head with an effect of 2.3. The critical value for inclusion in the regression model was a value of 1.99. All interactions and combinations of factors that achieved a standardized effect lower than the critical value were excluded from the regression analysis.

**Figure 11:**
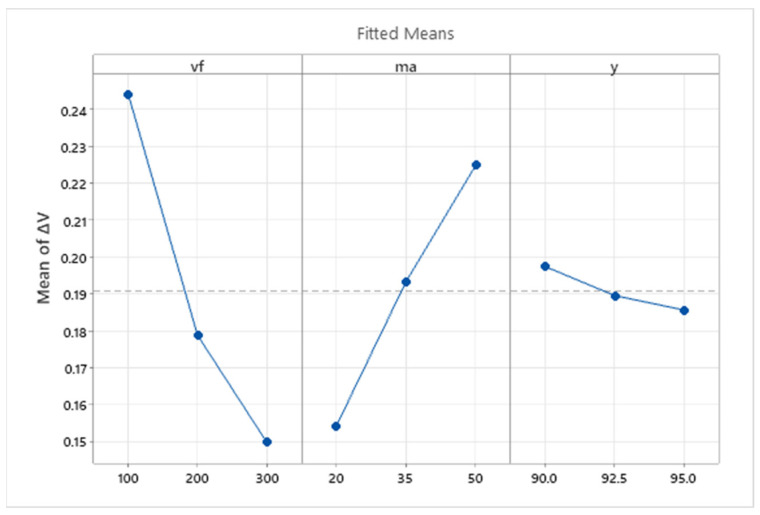
Main effect plot for Δ*V*.

The effect of the main technological conditions (Figure 11) on the response value shows the influence of individual factors on the obtained values of the monitored response Δ*V*. As can be seen from the graph, the speed of the dividing head has the greatest influence on the obtained value of the dependent variable. This effect has a negative trend, where with increasing the value of *v_f_*, there is a significant decrease in the monitored response Δ*V*. The opposite trend can be observed in the mass flow of the abrasive, where a positive effect is present, and the value of the monitored response increases with an increase in *m_a_*. The effect of the inclination angle of the cutting head *γ* is significant for the given regression model, based on Pareto analysis and variance analysis. With this factor, it is also possible to observe a negative effect on the monitored response Δ*V*.

**Figure 12:**
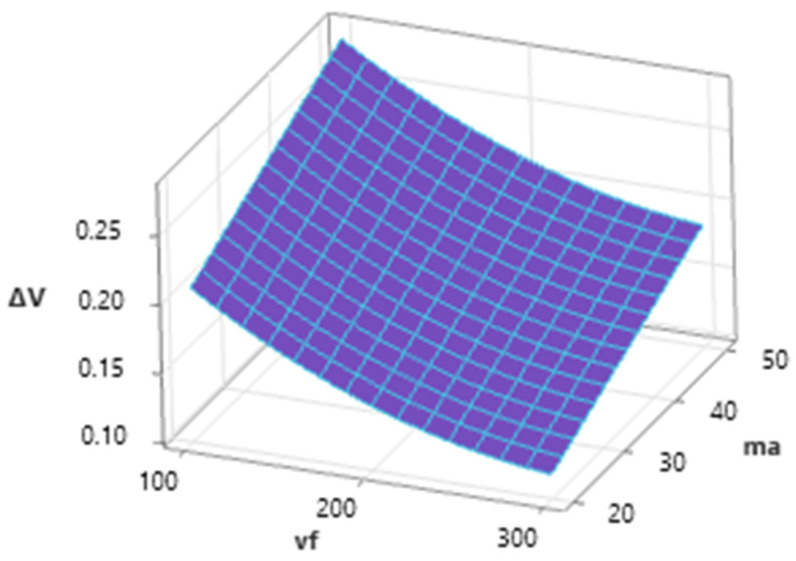
Dependence of Δ*V* on *v_f_* and *m_a_* (*γ* = 92.5°).

From the graph (Figure 12), it is possible to observe the influence of the two most significant technological conditions on the acquired values of the monitored response Δ*V*. Considering that *v_f_* and *m_a_* significantly affect the values of the dependent variable, the graph area has a decreasing trend in both axes, reaching from the maximum at *v_f_* = 100 mm.mn^−1^ and *m_a_* = 50 g·min^−1^ to the minimum at *v_f_* = 300 mm·min^−1^ and *m_a_* = 20 g·min^−1^.

**Figure 13:**
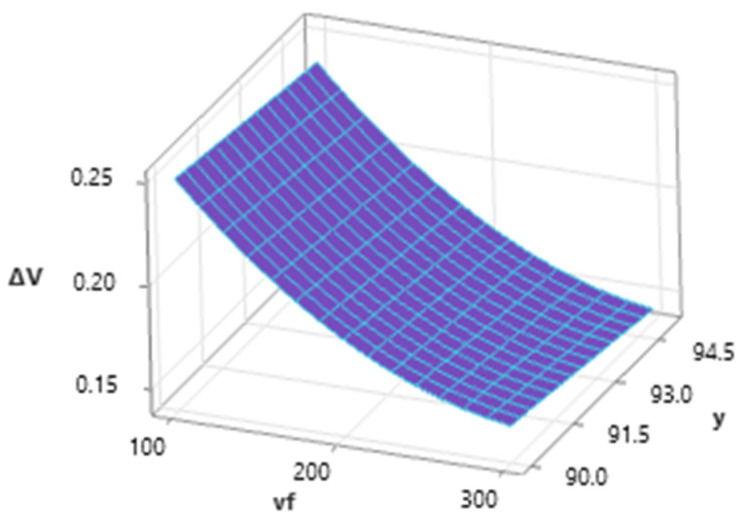
Dependence of Δ*V* on *v_f_* and *γ* (*m_a_* = 35 g·min^−1^).

The surface graph of the influence of *v_f_* and *γ* on Δ*V* (Figure 13) shows a significantly higher influence of the cutting head traverse speed in comparison with the inclination angle of the cutting head on the monitored response. The volumetric loss of the material decreases significantly with increasing the feed speed, and the surface of the graph slightly slopes towards the 95° inclination of the cutting head.

**Figure 14:**
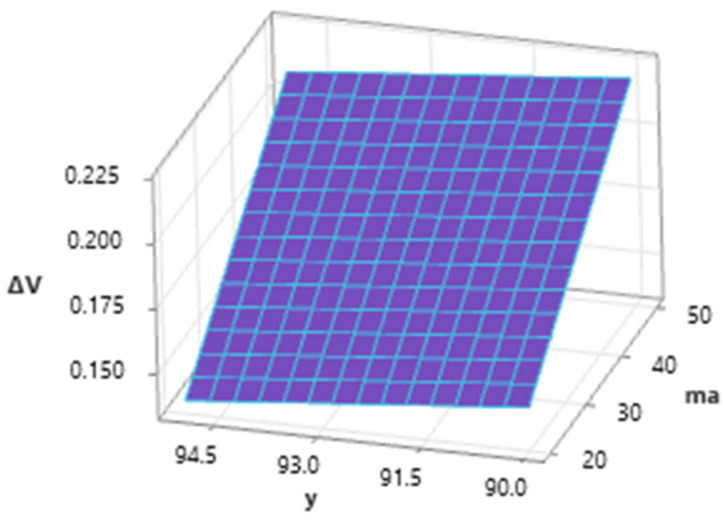
Dependence of Δ*V* on *γ* and *m_a_* (*v_f_* = 200 mm·min^−1^).

Analyzing the influence of the abrasive mass flow and the inclination angle of the cutting head (Figure 14), it is possible to state the high importance of ma on the overall value of the response in the form of volume loss of material. The influence of the inclination angle of the cutting head is also significant within the interaction with m_a_, and it is possible to observe a significant decrease in the values of the dependent variable when the inclination angle of the dividing head is changed by 90–95°.

**Figure 15:**
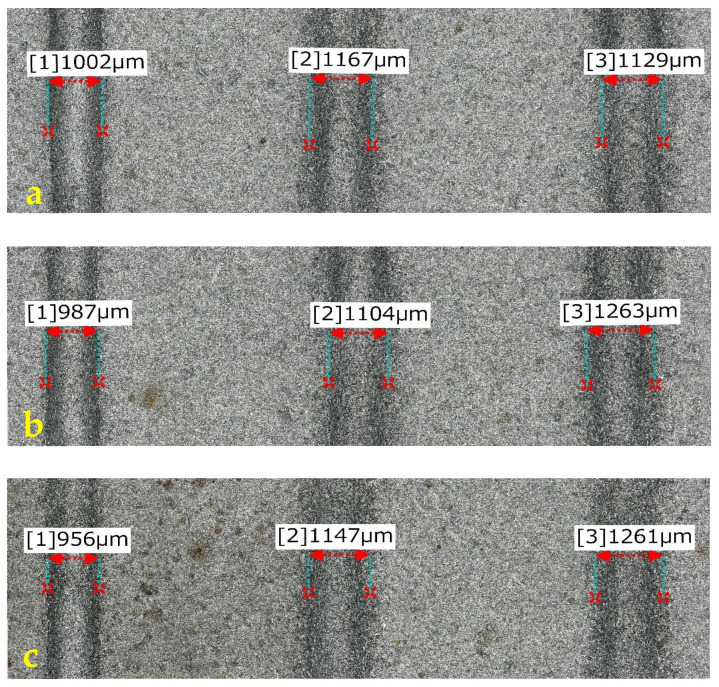
Width of erosion track.

The width of the erosion track (Figure 15) at the angle of the cutting head at *γ* = 90° and the abrasive mass flow in the range of 20–50 g·min^−1^ decreases from 1.002 mm to 0.956 mm (difference 46 µm). With a variation in the angle of inclination of the dividing head *γ* = 92.5° and 95°, it is possible to observe higher width values for all levels of *m_a_*, ranging from 1.104 mm at *m_a_* = 35 g·min^−1^ and *γ* = 92.5° to 1.261 at *m_a_* = 50 g·min^−1^ and *γ* = 92.5°, respesctively, 1.263 at *m_a_* = 35 g·min^−1^ and *γ* = 92.5°.

**Figure 16:**
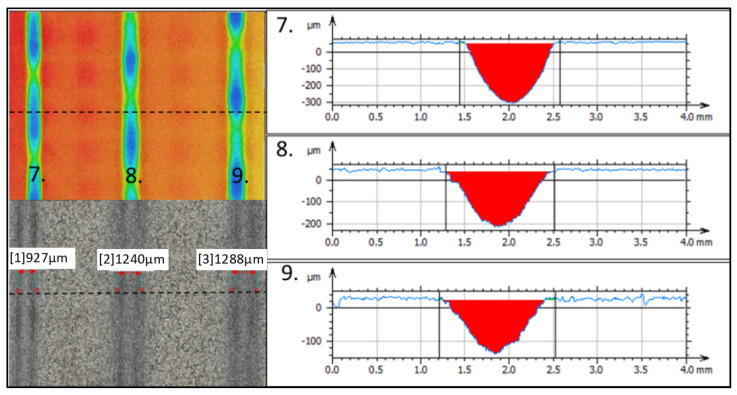
Maximal erosion effect.

Figure 15 shows the widths of the grooves created in the material Ti 6Al 4V created with one pass of AWJ with a traverse speed of the cutting head of 100 mm·min^−1^ (a) *m_a_* = 20 g·min^−1^ (from the left, *γ* = 90 °, 92.5°, and 95°), 1 (b) *m_a_* =35 g·min^−1^ (from the left *γ* = 90°, 92.5°, and 95°), and 1 (c) *m_a_* = 50 g·min^−1^ (from the left *γ* = 90°; 92.5° and 95°). The maximum erosion effect for Ti 6Al 4V (Figure 16) was observed at the combination of 7th technological parameters (*v_f_* = 100 mm·min^−1^; *m_a_* = 50 g·min^−1^; *γ* = 90°) at 365.4 µm with a width of 927 µm. Traces of erosion, created with the same combination of speed of the cutting head and abrasive mass flow, vary with the angle of the inclination of the cutting head, reaching a depth of 257.3 µm with a width of 1240 µm at *γ* = 92.5° and a depth of 164.9 µm and a width of *γ* = 95° 1288 µm.

**Figure 17:**
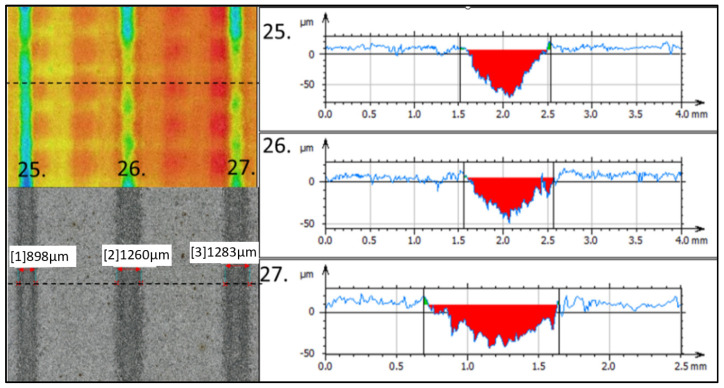
Minimal erosion effect.

The lowest value of the depth of removal for the material Ti 6Al 4V (Figure 17.) was recorded with a combination of 27 technological parameters (*v_f_* = 300 mm·min^−1^; *m_a_* = 50 g·min^−1^; *γ* = 95°) at the level of 54.48 µm with an erosion track width of 1283 µm. Adjacent erosion tracks formed at a smaller tilt angle of the cutting head reached a depth of 82.22 µm and a width of 898 µm at *γ* = 90° and a depth of 55.12 µm at a width of 1260 µm for *γ* = 92.5°.

## 4. Discussion

The DoE 3^3^ method was used for the planning of the experiment, and the evaluation was carried out based on analysis of variance (ANOVA) and regression analysis. Regression models were composed for the influence of selected technological parameters (traverse speed of cutting head, mass flow of abrasive and angle of inclination of the cutting head) at a low pressure of 50 MPa on the depth of the erosion track and volume loss of the material. Considering that AWJ-controlled depth machining is widely performed at lower pressures than material cutting, but typically using pressures in the 100–200 MPa range, there are few sources to compare the achieved results. Using variance analysis, the significance of the influence of individual technological parameters on the selected removal characteristics was determined. Furthermore, the width of the erosion track was evaluated as a stepping stone for planning further research in multi-pass machining when overlapping the track. The methods of optical profilometry and digital 3D microscopy were used for the evaluation of removal characteristics.

Based on the achieved results, it can be concluded that within the chosen level of technological parameters, the traverse speed of cutting head has the most significant influence on the removal characteristics, followed by the mass flow of the abrasive, and the angle of the inclination of the cutting head had the least influence in terms of the analyzed depth and volume loss. Conversely, the angle of the inclination of the cutting head has a significant influence on the width of the erosion track after the abrasive water jet, as a wider track is formed when the head is tilted. When a tilted cutting head is observed, the width of the track is wider in the range of 10–40% in comparison with the perpendicular position of the cutting head.

## 5. Conclusions

This paper focused on describing the controlled depth machining of titanium alloy Ti 6AL 4V, which shows higher values of mechanical properties in contrast with low density. In general, titanium alloys are considered hard-to-machine materials. Rising demands for the production of complex parts come with the necessity of finding new ways of machining. The application of a low-pressure abrasive water jet as a tool for machining seems to have potential. The absence of rigid tools, and thus the absence of tool wear, allows higher stability and sustainability of the manufacturing process. Also, the absence of thermal affection on a machined material is a great added value of water jet technology.

The presented research described the basic parameters of material removal to the design of the experiments (depth of cut a_p_, material removal rate ΔV, width of cut a_e_) within the machining using the AWJ at 50 MPa. Three factors were selected as independent variables, namely the traverse speed of the cutting head, abrasive mass flow, and tilt of the cutting head in three levels.

Main findings of presented research:

- In the selected range of technological parameters, their influence on the depth of the cut, width of the cut, and volume of material removal was described.
- A maximal erosion effect in terms of depth of cut was observed for *v_f_* = 100 mm·min^−1^, *m_a_* = 50 g·min^−1^, and *γ* = 90° at the level of 365.4 µm
- A minimal erosion effect in terms of depth of cut was observed for *v_f_* = 300 mm·min^−1^, *m_a_* = 50 g·min^−1^, and *γ* = 95° at the level of 54.48 µm
- A minimal width of cut a_e_ was observed for *v_f_* = 200 mm·min^−1^, *m_a_* = 50 g·min^−1^, and *γ* = 90° at the level of 870 µm
- A maximal width of cut a_e_ was observed for *v_f_* = 300 mm·min^−1^, *m_a_* = 35 g·min^−1^, and *γ* = 95° at the level of 1370 µm
- A maximal volume material removal Δ*V* was observed for *v_f_* = 100 mm·min^−1^, *m_a_* = 50 g·min^−1^, and *γ* = 90° at the level of 0.300 mm^3^·s^−1^
- A minimal volume material removal ΔV was observed for *v_f_* = 300 mm·min^−1^, *m_a_* = 20 g·min^−1^, and *γ* = 92.5° at the level of 0.078 mm^3^·s^−1^
- A regression equation for the depth of the cut and volume material removal was created.

Research in the field of AWJ-controlled depth machining still has potential in the machining of hard-to-machine materials. Using low pressure lowers the amount of water used in the process as well as energy consumption. The optimization of low-pressure machining thus not only brings the advantages of water jet technology, but also helps to reduce the need for resources, making production more sustainable. In the future, there is a need for the optimization of low-pressure AWJ machining, testing of multi-pass machining, and overlapping of jet paths. Emphasis should be placed on surface roughness parameters, surface integrity, and geometric stability of produced shapes.

## Figures and Tables

**Table 1:** Chemical composition and mechanical properties of Ti 6AL 4V.

Chemical Composition							
Element	Ti	Al	V	Fe	O	C	N
**%**	87.6–91	5.5–6.7	3.5–4.5	≤0.4	≤0.2	≤0.08	≤0.05
Mechanical properties							
	Density [kg·m^−3^]	Elongation [%]	Poisson ratio [-]	Tensile strength [MPa]	Yield point [MPa]		
	4430	18	0.30–0.33	895–1000	825–910		

**Table 2:** Fixed parameters of experiment.

Parameter	Value
*p*	50 MPa
*SOD*	4 mm
*Dv/Df*	0.33/1.02 mm

**Table 3:** Variable parameters of machining.

Parameter	Marking	Unit	Level 1	Level 2	Level 3
Traverse speed	*v_f_*	mm·min^−1^	100	200	300
Abrasives mass flow	*m_a_*	g·min^−1^	20	35	50
Tilt angle	*γ*		90	92.5	95

**Table 4:** Results of ANOVA for *a_p_*.

Term	Coef	SE Coef	T-Value	*p*-Value
Constant	810.9	72.2	11.23	0.000
*γ*	−4.399	0.769	−5.72	0.000
*m_a_*	1.162	0.128	9.07	0.000
*v_f_*	−2.366	0.135	−17.59	0.000
*v_f_* × *v_f_*	0.003792	0.000333	11.39	0.000

**Table 5:** Results of ANOVA for Δ*V_Ti_*.

Term	Coef	SE Coef	T-Value	*p*-Value
Constant	0.4805	0.0951	5.05	0.000
*v_f_*	−0.001196	0.000177	−6.75	0.000
*m_a_*	0.002353	0.000169	13.95	0.000
*γ*	−0.00235	0.00101	−2.32	0.023
*v_f_* × *v_f_*	0.000002	0.000000	4.13	0.000

## Data Availability

Data are contained within the article.
